# Quantitative diagnostic imaging of cancer tissues by using phosphor-integrated dots with ultra-high brightness

**DOI:** 10.1038/s41598-017-06534-z

**Published:** 2017-08-08

**Authors:** Kohsuke Gonda, Mika Watanabe, Hiroshi Tada, Minoru Miyashita, Yayoi Takahashi-Aoyama, Takashi Kamei, Takanori Ishida, Shin Usami, Hisashi Hirakawa, Yoichiro Kakugawa, Yohei Hamanaka, Ryuichi Yoshida, Akihiko Furuta, Hisatake Okada, Hideki Goda, Hiroshi Negishi, Kensaku Takanashi, Masaru Takahashi, Yuichi Ozaki, Yuka Yoshihara, Yasushi Nakano, Noriaki Ohuchi

**Affiliations:** 1 Department of Medical Physics, Graduate School of Medicine, Tohoku University, Seiryo-machi, Aoba-ku, Sendai 980-8575 Japan; 2 Department of Nano-Medical Science, Graduate School of Medicine, Tohoku University, Seiryo-machi, Aoba-ku, Sendai 980-8575 Japan; 3 Department of Pathology, Tohoku University Hospital, Seiryo-machi, Aoba-ku, Sendai 980-8574 Japan; 4 Department of Breast and Endocrine Surgical Oncology, Graduate School of Medicine, Tohoku University, Seiryo-machi, Aoba-ku, Sendai 980-8574 Japan; 5 Department of Gastroenterological Surgery, Graduate School of Medicine, Tohoku University, Seiryo-machi, Aoba-ku, Sendai 980-8574 Japan; 6 Department of Breast Surgery, Iwate Prefectural Central Hospital, Ueda, Morioka 020-0066 Japan; 7 Department of Breast Surgery, Tohoku Kosai Hospital, Kokubuncho, Aoba-ku, Sendai 980-0803 Japan; 8 Department of Breast Surgery, Miyagi Cancer Center Hospital, Medeshima, Natori 981-1293 Japan; 9 Department of Breast Surgery, Nihonkai General Hospital, Akiho-cho, Sakata 998-8501 Japan; 10 Department of Breast Surgery, Osaki Citizen Hospital, Furukawa, Osaki, 989-6174 Japan; 11 Department of breast surgery, Japanese Red Cross Ishinomaki Hospital, Ishinomaki, Miyagi 986-8522 Japan; 12 Bio Health Care, Business Development Division, Business Development Headquarters, Konica Minolta, Inc., No. 1, Sakura-machi, Hino-shi, Tokyo 191-8511 Japan; 13 Data Science Center, Systems Technology Division, Business Development Headquarters, Konica Minolta, Inc., No. 1 Sakura-machi, Hino-shi, Tokyo 191-8511 Japan

## Abstract

The quantitative sensitivity and dynamic range of conventional immunohistochemistry (IHC) with 3,3′-diaminobenzidine (IHC-DAB) used in pathological diagnosis in hospitals are poor, because enzyme activity can affect the IHC-DAB chromogenic reaction. Although fluorescent IHC can effectively increase the quantitative sensitivity of conventional IHC, tissue autofluorescence interferes with the sensitivity. Here, we created new fluorescent nanoparticles called phosphor-integrated dots (PIDs). PIDs have 100-fold greater brightness and a more than 300-fold greater dynamic range than those of commercially available fluorescent nanoparticles, quantum dots, whose fluorescence intensity is comparable to tissue autofluorescence. Additionally, a newly developed image-processing method enabled the calculation of the PID particle number in the obtained image. To quantify the sensitivity of IHC using PIDs (IHC-PIDs), the IHC-PIDs method was compared with fluorescence-activated cell sorting (FACS), a method well suited for evaluating total protein amount, and the two values exhibited strong correlation (R = 0.94). We next applied IHC-PIDs to categorize the response to molecular target-based drug therapy in breast cancer patients. The results suggested that the PID particle number estimated by IHC-PIDs of breast cancer tissues obtained from biopsy before chemotherapy can provide a score for predicting the therapeutic effect of the human epidermal growth factor receptor 2-targeted drug trastuzumab.

## Introduction

Determining target protein expression levels in tissues is important for elucidating disease-associated mechanisms and categorizing the response to molecular target-based drug therapies. Various methods, including enzyme-linked immunosorbent assay (ELISA), fluorescence-activated cell sorting (FACS), and matrix-assisted laser desorption/ionization (MALDI), have been developed to quantify protein expression, and each has specific advantages. For example, FACS is a highly suitable method for assaying the total amount of protein present in cells. However, these methods cannot simultaneously evaluate both cellular morphology and properties that depend on protein expression levels^1–5^. Immunohistochemistry (IHC) enables the evaluation of both cellular characteristics^6^. In conventional IHC used in pathological diagnosis in hospitals, after the incubation of tissue sections with a primary antibody and a biotin-labeled secondary antibody, streptavidin-conjugated horseradish peroxidase (HRP) reacts with the secondary antibody, and this is followed by a chromogenic reaction of 3,3′-diaminobenzidine (DAB) with HRP^7^. Therefore, in IHC with DAB (IHC-DAB), the intensity of the DAB staining depends on the enzymatic activity of HRP and is significantly influenced by the reaction time, temperature and HRP substrate concentration. Consequently, the quantitative sensitivity of IHC-DAB is low.

Fluorescent labels are effective for increasing the quantitative sensitivity of IHC because the intensity of fluorescent materials is proportional to the intensity of the photon excitation energy^7^. Additionally, fluorescent labeling in conjunction with a dark background produces an image with a high signal-to-noise ratio. In previous studies, a fluorescence imaging system was developed using Cy-5 tyramide for compartmentalized, automated, quantitative analysis of histological sections (AQUA)^8, 9^. This method improved the quantitative sensitivity relative to IHC-DAB. However, general organic fluorescent molecules, such as FITC, Alexa Fluor, and Cy-5, have disadvantages, owing to their poor photostability and interference from tissue autofluorescence. In addition, signal amplification in the AQUA method occurs via enzymatic activity. Therefore, the AQUA method may leave a margin for improvement to allow for IHC with high quantitative sensitivity in clinical practice.

Recently, various types of fluorescent nanoparticles have been developed, including commercially available quantum dots (Qdots)^10, 11^, which exhibit greater photostability and brightness than general organic fluorescent molecules. However, tissue autofluorescence is strong and has an intensity comparable to Qdot fluorescence intensity. Therefore, in the presence of tissue autofluorescence, quantitative IHC-based analysis using only the fluorescence intensity of Qdots is challenging. We have recently developed a quantitative IHC-based analysis using Qdots (IHC-QDs) with an autofluorescence-subtracted image and single-particle Qdot imaging^12, 13^. For IHC-QDs analysis, we created a new optical system that combined a spinning disk confocal unit, electron multiplier charge-coupled device (EM-CCD) camera, and image processing^12, 14–16^. This optical system can be applied to various types of Qdot imaging applications intended for tissues (e.g., *in vivo* imaging^14, 15^). However, the produced system was not versatile. Most laboratories and pathology departments have only a normal optical microscope with a CCD camera, which is not sufficiently sensitive to detect single-particle Qdots. In addition, commercially available streptavidin-conjugated Qdots possess only 5–10 streptavidin molecules on their surface, thereby suggesting a low reactivity of Qdots with biotin-labeled secondary antibodies. Thus, most scientists and pathologists have high expectations that the fluorescence intensity and reactivity of nanoparticles to target proteins could be enhanced significantly over the corresponding values for Qdots.

To substantially mitigate these problems of IHC with fluorescent nanoparticles by using a versatile optical system, we created organic fluorophore assembly-conjugated nanoparticles suitable for IHC called phosphor-integrated dots (PIDs). The PIDs possess three main properties: (I) 100-fold greater fluorescence intensity relative to Qdots; (II) high photostability, owing to the densely packed fluorophores obtained through networked chemical binding of the fluorophores; and (III) hyper-reactivity, owing to the 2,460-molecule streptavidin-supported surface structure, which provides a dynamic range more than 300-fold greater than that of Qdots. Additionally, we developed an image-processing method to calculate the particle number of PIDs on images acquired by using a versatile optical system. Using these three characteristics of PIDs and image processing, we developed a quantitative diagnostic imaging system to measure the expression levels of target proteins on tissues on the basis of the number of PID particles. In fact, when the quantitative sensitivity of IHC using PIDs (IHC-PIDs) was compared with that of FACS, the two methods exhibited strong correlation. Moreover, the IHC-PIDs enabled quantitative estimation of the expression level of human epidermal growth factor receptor 2 (HER2), which plays a crucial role in breast cancer. The HER2 imaging data revealed that the PID particle number provides a remarkably accurate score for predicting the effect of HER2-targeted molecular drug therapy, thus suggesting that IHC-PIDs can dramatically improve the diagnostic capability of various molecular-targeted drug therapies.

## Results

### Preparation and characterization of PIDs

**Figure 1:**
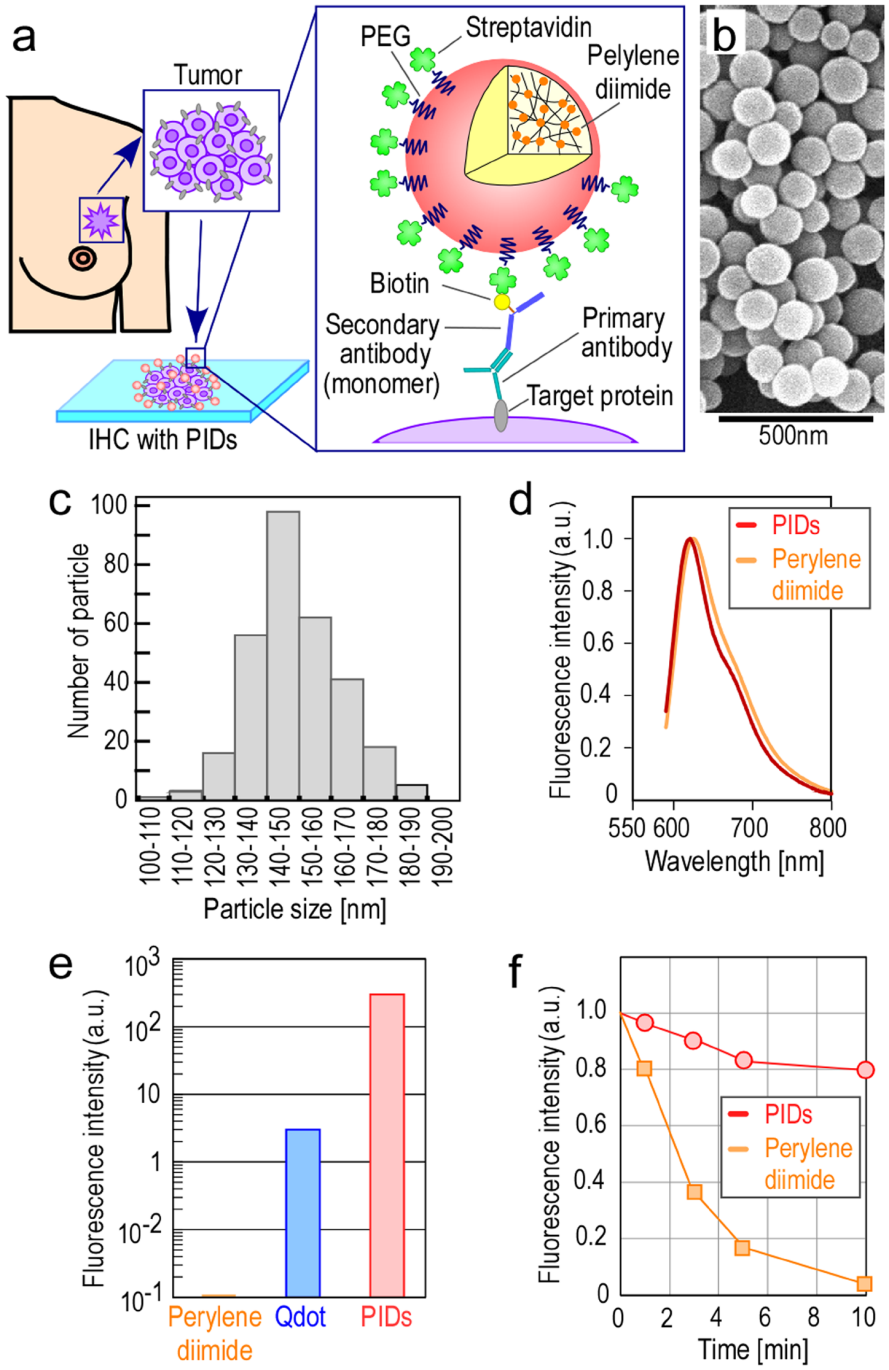
PID properties. (**a**) Schematic for the PID imaging of cancer tissues. We aimed to develop IHC with PIDs and measure the expression level of the target protein in cancer tissues with high accuracy. This schematic illustrates the histopathological diagnosis of breast cancer tissues excised from patients. The target proteins in cancer tissues were immunostained with a monoclonal primary antibody and a monoclonal secondary antibody that was monomerized and biotinylated, and then the samples were stained with PIDs coated with streptavidin by biotin-streptavidin binding. (**b**) SEM image of PID particles. Bar, 500 nm. (**c**) Distribution of PID particle sizes measured using SEM images. The average size was 149 nm (variation coefficient, 9%). (**d**) Fluorescence spectral patterns of the perylene diimide (orange line) and PID particles (red line). Both fluorescent materials were excited with a 580 nm laser and analyzed. (**e**) Comparison of 580 nm light-excited fluorescence intensities of perylene diimide, Qdots (Qdot 625), and PIDs. Each fluorescence intensity is shown on a logarithmic scale on the *y*-axis. (**f**) Comparison of the photostability of perylene diimide and PIDs. The perylene diimide or PIDs on the glass slide were irradiated by 580 nm light, and the fluorescence intensities of both fluorescent materials were measured over time. The fluorescence intensity of perylene diimide rapidly decreased to 2% over 10 min, whereas that of the PIDs remained at over 80% for the same irradiation condition.

We have previously developed organic fluorophore (tetramethylrhodamine)-assembly-conjugated nanoparticles (TMR nanoparticles) for IHC^17^. The fluorescence intensity of the TMR nanoparticles was 10-fold greater than that of the Qdots. However, because the fluorescence intensity of the TMR nanoparticles did not greatly exceed the tissue autofluorescence, we could not exclude the effect of autofluorescence interference^17^. In the present study, we prepared approximately 100,000 perylene diimide assembly-conjugated nanoparticles, called PIDs, for use in IHC (Fig. 1a). The PID particles were uniform in size (Fig. 1b) and had an average size of 149 nm (Fig. 1b,c). Fluorescence spectral analysis demonstrated that the 580 nm light-excited spectral patterns of the perylene diimide and PID particles peaked at approximately 620 nm (Fig. 1d). The 580 nm light-excited fluorescence intensity of the PID particles was approximately 100-fold greater than that of Qdot 625 (Fig. 1e), which exhibited fluorescence intensity comparable to the autofluorescence intensity. This result suggested that the PID fluorescence was significantly stronger than the tissue autofluorescence. The PIDs also possessed much greater photostability than that of perylene diimide (Fig. 1f). The 580 nm light-excited fluorescence intensity of perylene diimide rapidly decreased to 2% over 10 min, whereas the fluorescence intensity of the PIDs remained at a value greater than 80% under the same irradiation conditions. The photostability of the PIDs is attributable to the dense packing of the perylene diimide, owing to both the networked chemical binding of perylene diimide and the use of a capsule to protect the perylene diimide from oxidization. Moreover, a single PID has approximately 2,460 streptavidin molecules linked via polyethylene glycol (PEG) chains on its surface (Fig. 1a and Supplementary Fig. 1), whereas a single streptavidin-conjugated Qdot possesses only 5–10 streptavidin molecules. Therefore, PIDs are expected to exhibit significantly higher reactivity to the secondary antibody. In addition, although the PIDs have many streptavidin groups on the surface of a single nanoparticle, the density of streptavidin on the PIDs surface is comparable to that of streptavidin-conjugated Qdots. There are varieties of Qdots that possess different emission properties from short (e.g. Qdot 525) to long wavelength (e.g. Qdot 655). The Qdot number indicates the emission wavelength in nm. The emission wavelength of a Qdot shifts from short to long wavelength with increasing particle size. The form and size of Qdot 525 and Qdot 655 excluding surface PEG chains were observed by transmission electron microscopy^18^. From this information, we estimated the surface area of Qdot 525 and Qdot 655 to be 58.1 nm^2^ and 349.6 nm^2^, respectively. Streptavidin-conjugated Qdots have 5–10 streptavidin molecules linked via PEG chains on their surface. Thus, in Qdot 525 and Qdot 655, when five streptavidin molecules are conjugated, the density of streptavidin in a single Qdot is one streptavidin molecule per approximately 11.6 to 69.9 nm^2^. Similarly, when ten streptavidin molecules are conjugated, the density of streptavidin in a single Qdot is one streptavidin molecule per approximately 5.8 to 35.0 nm^2^. The total range of area for a single streptavidin molecule in Qdot 525 and Qdot 655 is about 5.8 to 69.9 nm^2^. On the other hand, the surface area of a single PID is 70,650 nm^2^. Since the number of streptavidin molecules in the PID is 2,460, the density of streptavidin in a single PID is one streptavidin molecule per approximately 28.7 nm^2^, falling in the middle of the range calculated for Qdot 525 and Qdot 655. Thus, the streptavidin density in PIDs is comparable to that of established technology.

### Development of an image-processing method to measure the number of PID particles

**Figure 2:**
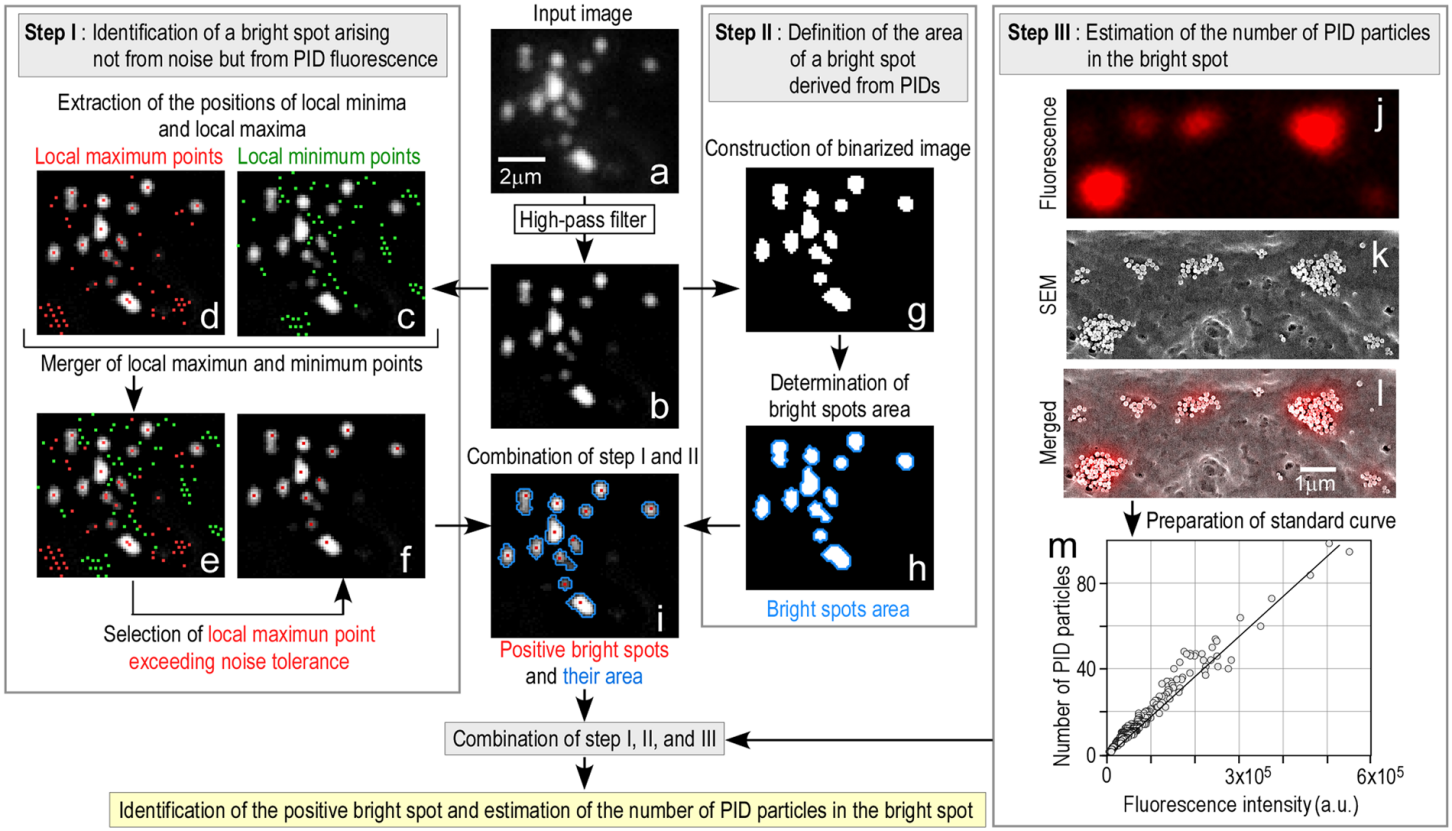
Image-processing method to measure the number of PID particles in an acquired image. This imaging-processing method consists of three main steps: (1) identification of a bright spot arising not from noise but from PID fluorescence (Step I), (2) definition of the area of a bright spot derived from PIDs (step II), and (3) estimation of the number of PID particles in the bright spot (step III). First, the input fluorescence image (**a**) was filtered with a high-pass filter (**b**). In step I, the high-pass-filtered image was scanned using a matrix filter of 3 × 3 pixels (Fig. 3a–c) to extract the positions of local minima (**c**, green dots) and local maxima (**d**, red dots). Then, a merged image was formed that showed the positions of both types of points (**e**). The region around each local maximum point was searched for the nearest local minimum point (Fig. 3d). We selected the local maximum point exceeding the “noise tolerance (Fig. 3d)” as a positive bright spot (**f**). The “noise tolerance” is a threshold value that shows that the brightness value of a local maximum point is higher than 32 (in the 12-bit image) relative to that of the corresponding local minimum point (Fig. 3d). In step II, a binarized image (**g**) was constructed from (**b**) by using a threshold value of 16 (in the 12-bit image), and then the bright spot area was determined (**h**, blue line) from the binarized image (**g**). The positive bright spots acquired in step I (**f**, red dots) and the bright area acquired in step II (**h**, blue line) were combined (**i**). Bar, 2 μm (**a**–**i**). In step III, the positive bright spots resulting from PIDs in human cancer tissue and their area were simultaneously imaged using fluorescence microscopy (**j**) and SEM (**k**). The patterns resulting from many positive bright spots (n = 443) were analyzed using both imaging methods (**l**). The data of both the fluorescence signals (**j**) and the particle number determined using SEM images (**k**) were combined (**l**), and a standard curve with high correlation (correlation coefficient, R = 0.98) was obtained (**m**). By combining steps I, II, and III, we identified positive bright spots and estimated the PID particle number per bright spot. Bar, 1 μm (**j**–**l**).

Our image-processing method included three steps: (step I) identification of a bright spot arising from PID fluorescence and not from noise; (step II) definition of the area of a bright spot derived from PIDs; and (step III) estimation of the number of PID particles in the bright spot. Prior to steps I–III, the input fluorescence image (Fig. 2a) was high-pass filtered to remove autofluorescence and noise (Fig. 2b) because the bright spot of the PID fluorescence contained more high-frequency components than the background fluorescence and noise.

**Figure 3:**
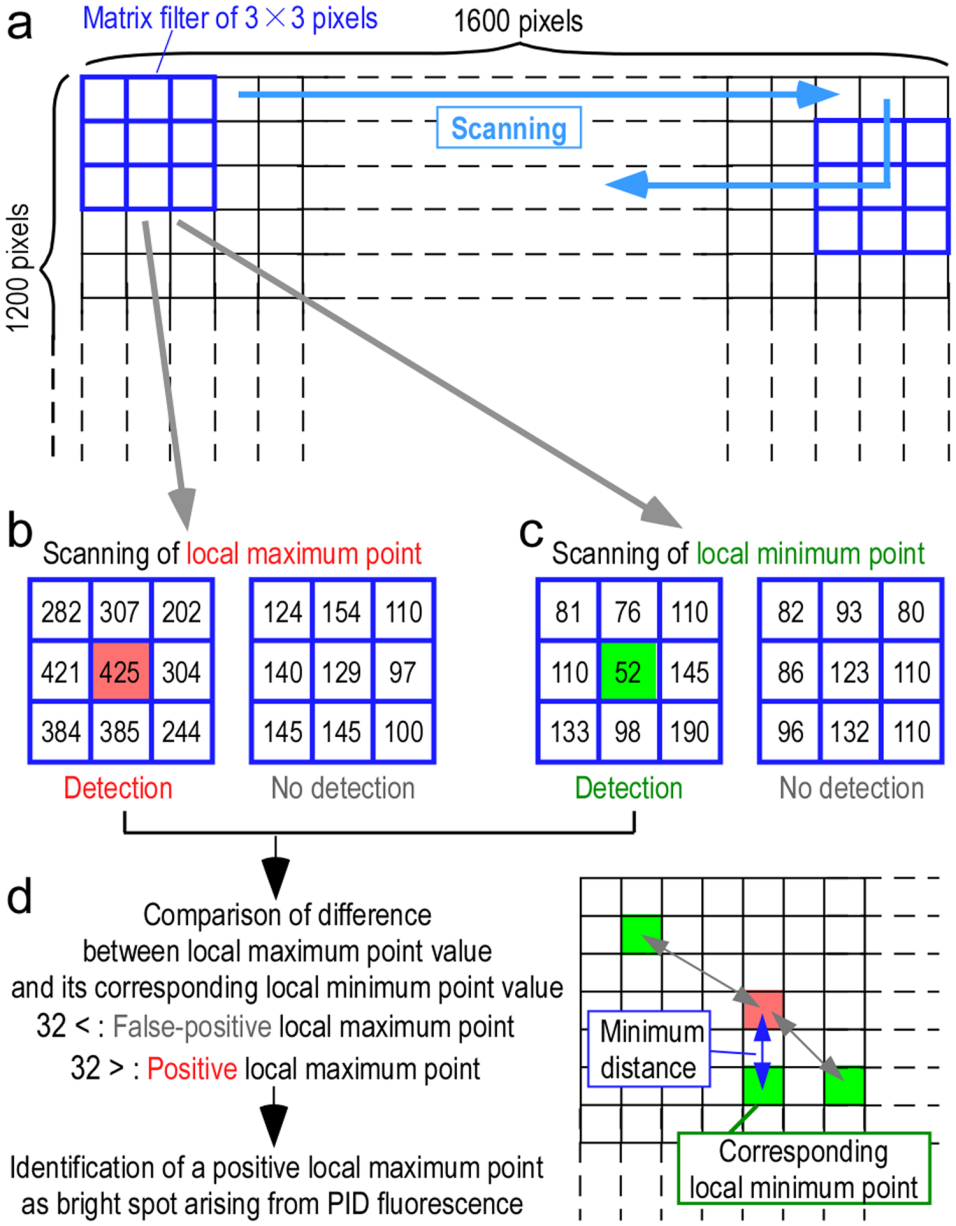
Identification of a positive local maximum point as a bright spot arising from PID fluorescence. All regions of the high-pass-filtered image were scanned using a matrix filter of 3 × 3 pixels to identify local maximum and minimum points (**a**). The local maximum points were identified in cases in which the center pixel value was higher than the 8 surrounding pixel values (**b**, red square). Similarly, the local minimum points were identified in cases in which the center pixel value was lower than the 8 surrounding pixel values (**c**, green square). In (**b**) and (**c**), the numerical values show the brightness value of each pixel in 12 bits. Therefore, no local maximum points or minimum points were observed when the distribution of the brightness values gradually changed (right panels in **b** and **c**). After all of the local maximum and local minimum points were merged into one image (Fig. 2e), the region around each local maximum point was searched for the nearest local minimum point. This point was called the corresponding local minimum point (**d**). The difference between the brightness of the local maximum point value and its corresponding local minimum point was determined for comparison. If the brightness value difference between the two point values was less than 32 (in the 12-bit image), the local maximum points were ignored as false-positive local maximum points (**d**). In contrast, the local maximum points that had brightness values greater than 32 (in the 12-bit image) were considered positive bright spots that resulted from PIDs (**d**).

First, in step I, all regions of the high-pass-filtered image (1,600 × 1,200 pixels) were scanned using a matrix filter of 3 × 3 pixels to identify a local maximum point (Figs 2d and 3a). Local maximum points were identified when the center pixel value was higher than the 8 surrounding pixel values (Figs 2d and 3b). Similarly, local minimum points were identified when the center pixel value was less than the 8 surrounding pixel values (Figs 2c and 3c). Therefore, no local maximum points or minimum points were observed when the distribution of the brightness values gradually changed (Fig. 3b,c). A merged image was created that showed both types of points (Fig. 2e). To identify the local maximum point that resulted from the bright spot of the PID fluorescence, the region around each local maximum point was searched for the nearest local minimum point (Fig. 3d). The difference between the brightness of the local maximum point value and its corresponding local minimum point was determined as a comparison (Fig. 3d). If the brightness value difference between the local maximum point and the corresponding local minimum point was less than 32 (in the 12-bit image), the local maximum points were ignored as false-positive points (Fig. 3d). We called the threshold value the “noise tolerance”. The local maximum points over the noise tolerance were adopted as bright spots that resulted from PIDs (Figs 2f and 3d). By properly setting the noise tolerance, we confirmed that the positive bright spots resulting from the PIDs were detected in fluorescence microscopy images with high accuracy (F-measure, F = 0.90).

Second, in step II, the high-pass-filtered images were binarized using a threshold of 16 (in the 12-bit image) (Fig. 2g), and the bright spot areas were determined (Fig. 2h). The images obtained in steps I and II were integrated (Fig. 2i) to determine positive spots that resulted from the PID signal and their areas. If the spots observed in Fig. 2h did not exist in the positive points identified in Fig. 2f, the bright spot area was ignored. The brightness values of the positive bright spots in Fig. 2i were then converted to the number of PID particles in the next step (step III).

Third, in step III, to investigate the relationship between the fluorescence intensity and the particle number in a bright spot, we simultaneously imaged PID bright spots in human cancer tissues using fluorescence to measure the fluorescence intensity of each bright spot (Fig. 2j,l) and scanning electron microscopy (SEM) to count the PID particle number of each bright spot (Fig. 2k,l). Various patterns derived from many positive bright spots (n = 443) were analyzed using both imaging methods. The fluorescence signals (Fig. 2j) were analyzed using the same method as in steps I and II, and then the particle number was determined using SEM images (Fig. 2k). Both scores were used to generate a standard curve (Fig. 2m) with a high correlation between the fluorescence signals and the PID particle number (correlation coefficient, R = 0.98). The combination of steps I, II, and III enabled the identification of positive bright spots and the estimation of the number of PID particles per bright spot.

### Examination of the dynamic range and quantitative sensitivity of IHC-PIDs

**Figure 4:**
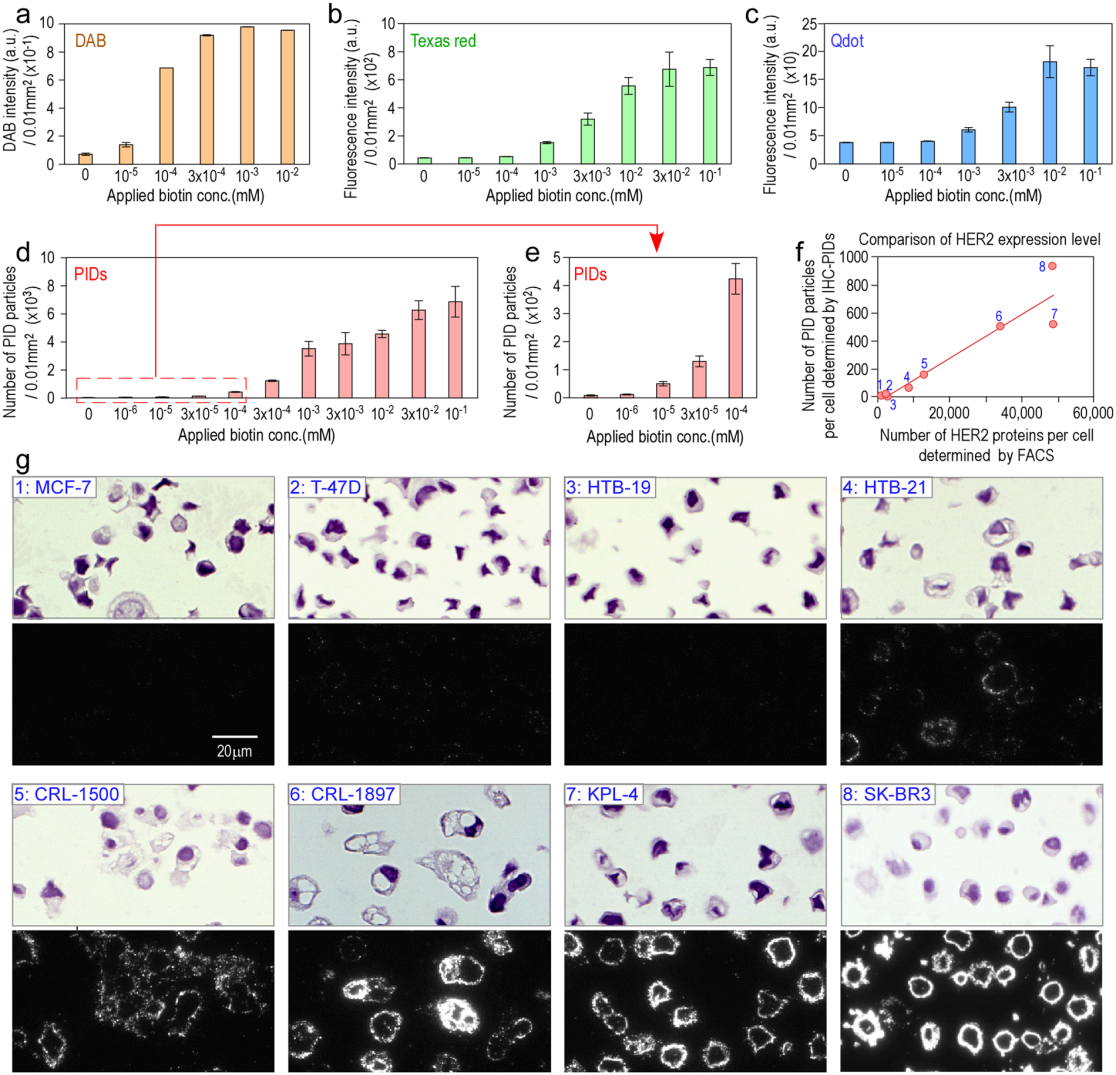
Dynamic range and quantitative sensitivity of IHC-PIDs. The sensitivity of imaging with PIDs (**d**,**e**) was compared with that with DAB (**a**), Texas Red (**b**), and Qdot 625 (**c**) using deparaffinized paraffin sections of human breast cancer tissues incubated with biotin solutions of varying concentration. The effective dynamic ranges of DAB (**a**), Texas red (**b**), Qdots (**c**), and PIDs (**d**,**e**) staining were 10^−5^ to 10^−3^, 10^−4^ to 3 × 10^−2^, 10^−4^ to 10^−2^, and 10^−6^ to 10^−1^ mM, respectively. Error bars indicate s.e.m (**a**–**e**). Next, cultured cells of eight types of breast cancer that express HER2 at various levels were stained with PIDs (**g**) to investigate whether IHC-PIDs can accurately measure the protein expression level in cultured cancer cells. These cells were immunostained with monoclonal anti-HER2 antibody, biotinylated monoclonal secondary antibody, and PIDs and were observed using a versatile fluorescence microscope. The images of all cells are displayed at the same magnification. Bar, 20 μm. The obtained fluorescence data were analyzed by image-processing method, shown in Figs 2 and 3, to determine the number of PID particles per cell (**f**, *y*-axis). Typical imaging data of these cells with PIDs are shown in (**g**). The HER2 expression levels of these cells were examined using FACS. We determined the mean number of HER2 proteins per cell using a standard curve and a kit for FACS (**f**, *x*-axis). The mean value of the PID particle number per cell (**f**, *y*-axis) was compared with the mean value of HER2 proteins per cell (**f**, *x*-axis), as determined by FACS. The two values were strongly correlated (R = 0.94) (**f**). The sample number in the graph (**f**) corresponds to the cancer cell image in (**g**).

Various concentrations of biotin solution were applied to a deparaffinized section of human breast cancer tissue to prepare a mimic slide. After incubation with biotin molecules, the non-bonded free biotin on the mimic slides was washed off. Streptavidin-conjugated HRP, streptavidin-conjugated Texas Red, and streptavidin-conjugated Qdots or PIDs were reacted with the biotin-bonded slide glass and observed. Streptavidin-conjugated HRP was visualized with DAB and analyzed with an Aperio image analysis system (Leica) to quantify the degree of brown coloring from IHC-DAB. Other samples were observed using a versatile fluorescence microscope with a normal CCD camera. The effective dynamic ranges of DAB, Texas Red, and Qdot staining were 10^−5^ to 10^−3^ (Fig. 4a), 10^−4^ to 3 × 10^−2^ (Fig. 4b), and 10^−4^ to 10^−2^ mM (Fig. 4c), respectively. By contrast, the effective dynamic range of the PIDs was 10^−6^ to 10^−1^ mM (Fig. 4d,e). Thus, of all the staining methods, PID staining produced the broadest dynamic range, from very low to fairly high. In addition, on the mimic slides incubated only with phosphate-buffered saline (PBS) lacking biotin (Fig. 4e, 0 mM condition), the PID particle number was 8.8 in 0.01 mm^2^ (100 μm × 100 μm square). The size of a cell is defined as a 10-μm square, so there are 100 cells in the 100 μm × 100 μm area. Therefore, an 8.8 PID particles in 0.01 mm^2^ (Fig. 4e, 0 mM condition) corresponds to about 0.09 PID particles in a cell. These data indicate that non-specific binding of PIDs to cancer tissues is very weak.

Next, the measurement quantitative sensitivity of IHC-PIDs was investigated using cultured cells of eight types of breast cancer. First, the HER2 expression levels of these cells were examined using FACS. FACS is not suitable for diagnosis of pathology specimens for which observation of cellular morphology and characteristics is needed. However, because FACS uses a small fluorescent dye molecule, among existing techniques it is a highly suitable method for assaying the total amount of proteins present in fixed cells. Consequently, the correlation with FACS was used to determine whether our technique was capable of assaying protein expression level in a pathology specimen with high accuracy. Each type of cell was fixed with formalin and then labeled with an anti-HER2 antibody and a secondary antibody conjugated to Alexa Fluor 488. We measured the fluorescence intensity per cell by FACS (n = 20,000)^19^ and then calculated the mean number of HER2 proteins per cell by using a standard curve. SK-BR3 cells exhibited the highest expression level of HER2, with approximately 5 × 10^4^ proteins per cell (Fig. 4f, *x*-axis). These results suggest that the actual number of HER2 proteins that can be recognized by the anti-HER2 antibody is approximately 5.0 × 10^4^, owing to the effect of formalin fixation, although the number of HER2 proteins in living SK-BR3 cells is known to be 2.0 × 10^6^ per cell^20^. Next, paraffin sections of these cultured cells were immunostained with a monoclonal anti-HER2 antibody and a monomerized and biotinylated monoclonal secondary antibody, and PIDs and were observed using a versatile fluorescence microscope (Fig. 4g). The obtained fluorescence data were analyzed via the newly developed image-processing method (Figs 2 and 3) to determine the number of PID particles per cell. The mean number of PIDs per cell (Fig. 4f, *y*-axis) was then calculated and compared with the value determined by FACS (Fig. 4f, *x*-axis). The two values exhibited strong correlation (R = 0.94) (Fig. 4f), thus demonstrating that IHC-PIDs can measure the protein expression level in cells with high accuracy.

### Application of IHC-PIDs to the categorization of the response to HER2-targeted drug therapy

**Figure 5:**
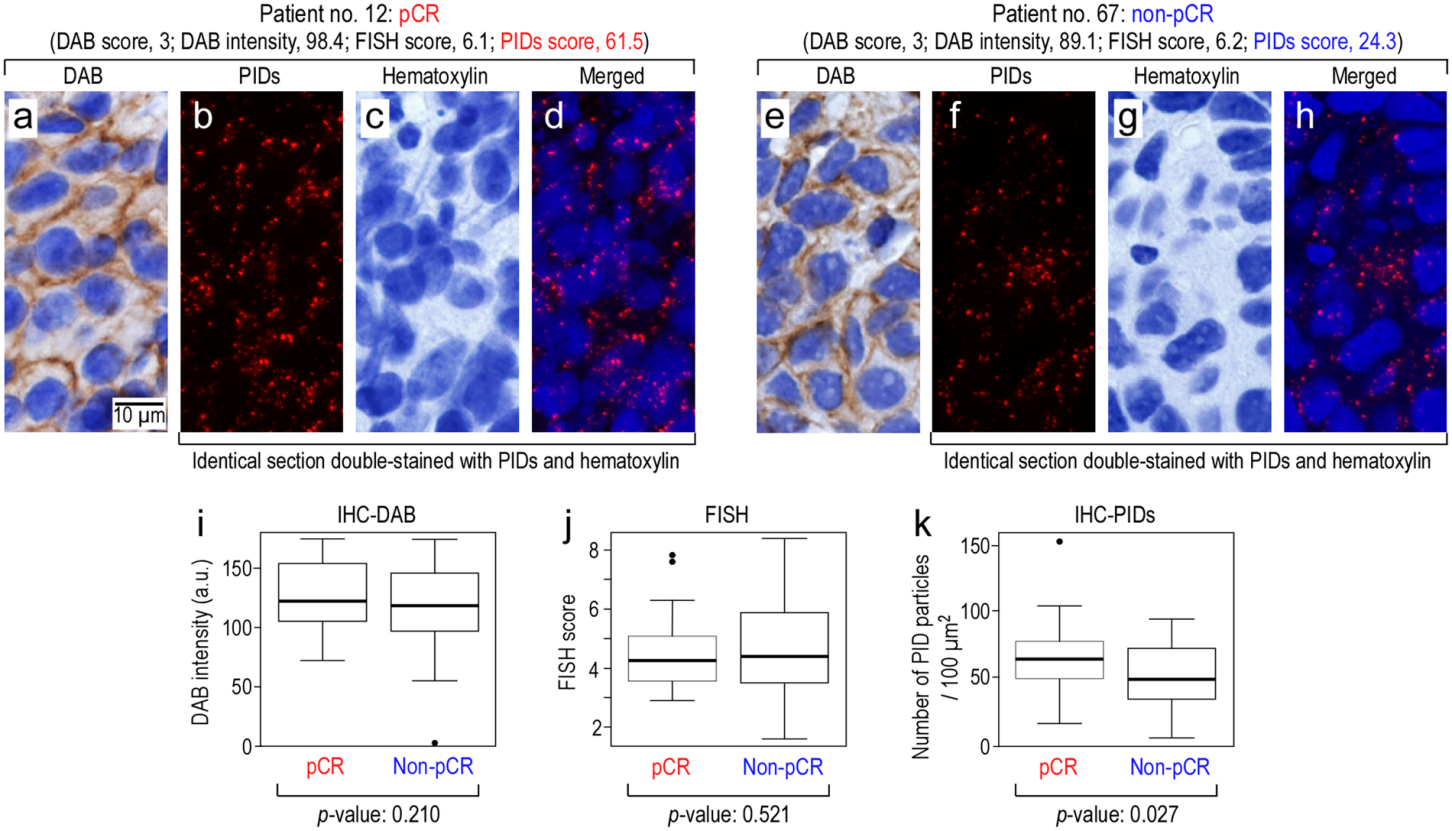
Categorization of the response to anticancer drug therapy using IHC-PIDs. We acquired tissues from pre-chemotherapy biopsies from 74 HER2-positive patients (Supplementary Table 1). These tissues were tested with IHC-DAB (**a**,**e**), FISH or IHC-PIDs (**b**–**d**, **f**–**h**). (**a**–**d**) and (**e**-**h**) show typical samples of patients 12 and 67, respectively, as numbered in Supplementary Table 1. Both tissues have a score of 3 in HER2 testing by IHC-DAB and a FISH score of approximately 6. DAB intensity of patient no. 12 (score, 98.4) and no. 67 (score, 89.1) analyzed using Aperio image analysis were similar (**a**,**e**). However, patient no. 12 had a pCR, and patient no. 67 had a non-pCR. (**a**), (**b**–**d**), (**e**) and (**f**–**h**) represent different sections. (**b**–**d**) and (**f**–**h**) are identical sections double-stained with PIDs and hematoxylin, respectively. The images of all tissues samples are displayed at the same magnification. Bar, 10 μm. These staining results show that IHC-DAB and FISH could not distinguish between pCR (n = 34) and non-pCR (n = 40). The *p*-values of the *t*-tests for IHC-DAB and FISH were 0.210 (**i**, boxplot) and 0.521 (**j**, boxplot), respectively, and did not indicate significant differences. By contrast, the *p*-value of the *t*-test for the IHC-PIDs was 0.027, and this imaging clearly distinguished pCR (n = 34) from non-pCR (n = 40) (**k**, boxplot).

Trastuzumab is a humanized monoclonal antibody against HER2^21^. In clinical practice, HER2 gene amplification and protein overexpression have been measured by fluorescence *in situ* hybridization (FISH) and IHC-DAB. IHC-DAB against HER2 is classified into only 4 categories (scores of 0, 1, 2, and 3); furthermore, these categories are not based on quantitative amounts of HER protein. According to the recommended practice guideline for HER2 testing, negative for HER2 is defined as IHC-DAB scores of 0–1+, equivocal for HER2 is defined as IHC-DAB scores of 2+, and positive for HER2 is defined as IHC-DAB scores of 3+. In cases of score 2+, FISH is required to judge whether HER2 positive or negative^22^. Recently, neoadjuvant chemotherapy with trastuzumab has been administered to HER2-positive patients^23^. When the HER2 status of breast cancer tissues obtained from biopsy before chemotherapy and surgery is categorized as score 3 in HER2 testing by IHC-DAB or over score 2.0 in HER2 testing by FISH, anthracycline, a topoisomerase inhibitor, is administered for three months and is followed by trastuzumab and taxanes, paclitaxel, or docetaxel, which are microtubule inhibitors, for three months^24^. After treatments, if surgically removed cancer tissues do not exhibit invasive cancer cells and lymph-node metastasis, the patients are defined as having a pathologic complete response (pCR)^25^. By contrast, if the cancer tissues have invasive cancer cells and/or lymph-node metastasis, the patients are defined as having a non-pathologic complete response (non-pCR)^25^. We acquired tissues obtained from biopsy before chemotherapy from 74 HER2-positive patients who received neoadjuvant chemotherapy (Supplementary Table 1). These tissues were examined by IHC-DAB (Fig. 5a,e), FISH or IHC-PIDs (Fig. 5b–d,f–h). The staining intensity of IHC-DAB was analyzed using Aperio image analysis (Fig. 5i). The tissues of patient nos. 12 and 67 both had a HER2 score of 3 by IHC-DAB and a FISH score of approximately 6. The DAB intensities of patient no. 12 (score, 98.4) and no. 67 (score, 89.1) analyzed using Aperio image analysis were similar. However, patient no. 12 had pCR, and patient no. 67 had non-pCR. The staining results from 74 HER2-positive patients suggest that IHC-DAB (Fig. 5i, *p* = 0.210) and FISH (Fig. 5j, *p* = 0.521) were unable to distinguish between cases of pCR (n = 34) and non-pCR (n = 40). By contrast, IHC-PIDs distinguished pCR (n = 34) from non-pCR (n = 40) with statistical significance (*p* = 0.0272) (Fig. 5k).

### Versatility of IHC-PIDs

**Figure 6:**
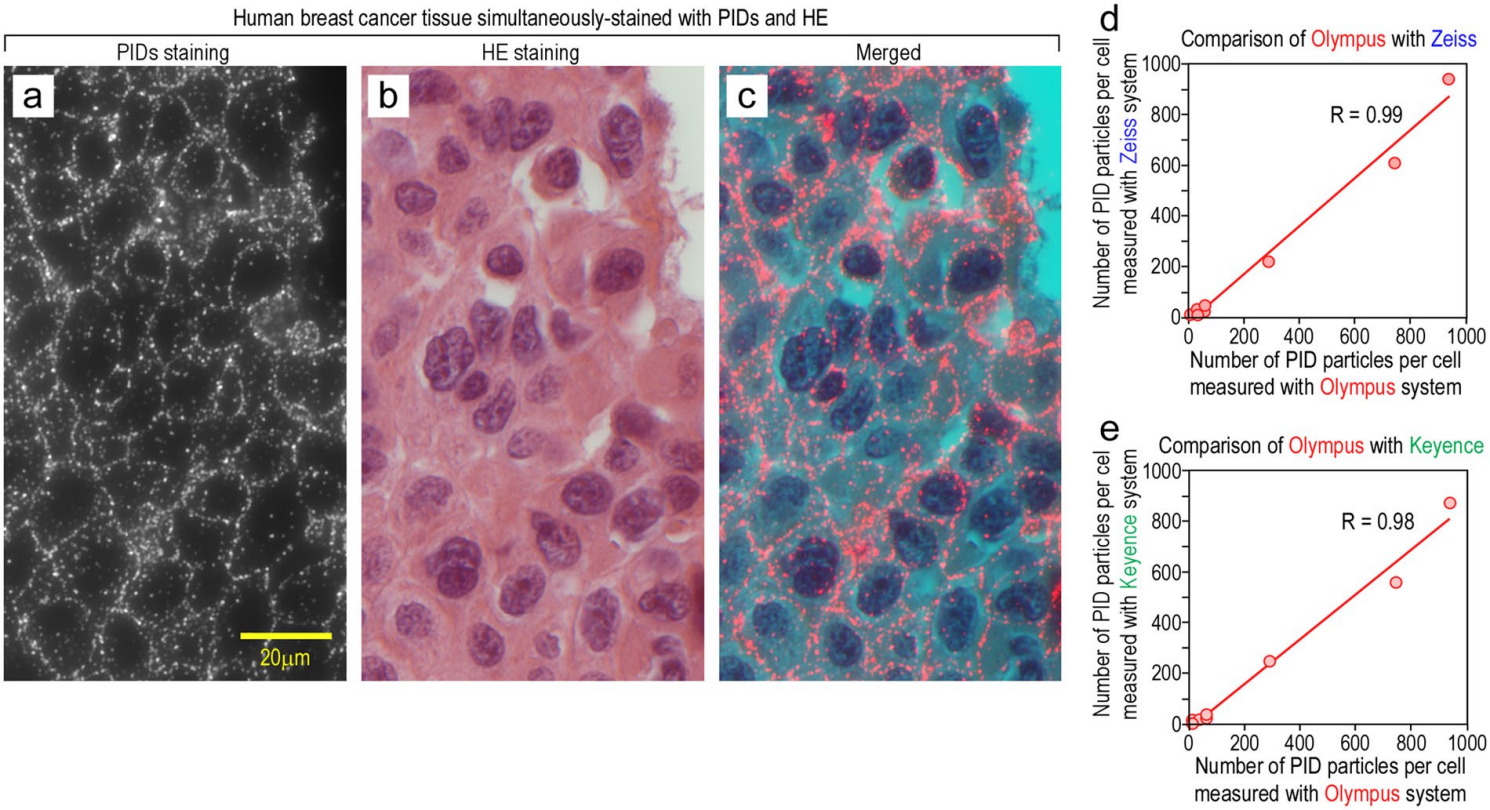
Examination of versatility of IHC-PIDs. (**a**–**c**) Double staining of cancer tissue with PID and HE. The samples were immunostained with PIDs (**a**) and then stained with HE (**b**). In previous studies, double staining using fluorescence and eosin has been challenging because the fluorescence intensity of eosin is relatively high. However, the fluorescence intensity of the PIDs was higher than that of eosin. Thus, PID staining enabled us to simultaneously observe both the cellular morphology and the cellular properties using a single tissue section (**c**). Bar, 20 μm. (**d**,**e**) To provide more evidence of the versatility of IHC-PIDs, we evaluated the correlation between data obtained using fluorescence microscopes from the Olympus, Zeiss, and Keyence corporations. HER2 expression in cultured cells of eight types of breast cancer, shown in Fig. 4g, was quantified by the number of PID particles per cell using Zeiss and Keyence fluorescence microscopes. We then evaluated the correlation between the data obtained using fluorescence microscopes from Olympus and Zeiss (**d**) and Olympus and Keyence (**e**). The data obtained using the Zeiss and Keyence microscopes were significantly correlated with the Olympus data (both with correlation coefficients R > 0.98).

In clinical practice, hematoxylin-eosin (HE) staining is the most widely used method to visualize cellular morphology, and pathologists use information about cellular morphology to diagnose cancer cell behavior, on the basis of their clinical experience. Therefore, to perform pathological diagnosis with high accuracy, simultaneous observation of both cellular morphology and properties using a single tissue section would be beneficial. However, simultaneous observation of these properties is difficult for the following two reasons: (I) the brown color of DAB staining is similar to the red color of eosin staining, thus hindering their differentiation; and (II) the fluorescence intensity of eosin is far superior to that of Qdots, thus making it difficult to observe a fluorescent dye or Qdot on an eosin-stained sample. However, the fluorescence intensity of PIDs is higher than that of eosin. Thus, we detected the PID signal (Fig. 6a,c) on samples stained with HE (Fig. 6b,c) and clearly observed the atypical nuclei and cell shapes. Therefore, this method enabled us to evaluate both the cellular morphology and properties in a single tissue sample.

In this study, IHC-PIDs analysis of cultured human breast cancer cells and tissues was performed using a fluorescence microscope manufactured by the Olympus Corporation. To provide more evidence of the versatility of IHC-PIDs, we sought to perform PID imaging using versatile microscopes from other companies. We measured the HER2 expression levels of eight types of cultured cancer cells, shown in Fig. 4g, as the number of PID particles number per cell by using fluorescence microscopes from Zeiss and Keyence. Then, the correlation between the data obtained using fluorescence microscopes from Olympus and Zeiss (Fig. 6d) and Olympus and Keyence (Fig. 6e) was evaluated. For these measurements, a BX53 fluorescence microscope (Olympus) with a DP73 CCD camera (Olympus), an Axio Imager Z1 fluorescence microscope (Zeiss) with an AxioCam CCD camera (Zeiss), and a BZ-X700 fluorescence microscope (Keyence) with a BZ-X710 CCD camera (Keyence) were used. The data obtained using the Zeiss (Fig. 6d) and Keyence (Fig. 6e) microscopes were significantly correlated with the data for the Olympus microscope (both with correlation coefficients R > 0.98), thus demonstrating that the IHC-PIDs method enables accurate measurement of protein expression levels independently of the optical system.

## Discussion

As part of interdisciplinary translational research integrating nanotechnology and clinical pathology, in this study we created new fluorescent nanoparticles called PIDs that have 100-fold greater fluorescence intensity than that of Qdots. Qdot brightness is not sufficient to completely avoid interference from autofluorescence because of the comparable intensity of tissue autofluorescence. In contrast, PID fluorescence was significantly stronger than the tissue autofluorescence and was able to produce an image with a high signal-to-noise ratio in the presence of autofluorescence.

A newly developed image-processing method enabled the calculation of the PID particle number in a bright spot of the obtained image by using a versatile optical system with high accuracy. Therefore, our IHC-PIDs method can identify a single PID that recognizes a target molecule quantitatively in IHC under a versatile optical system. Importantly, the fluorescence signal should be measured not as the PID fluorescence intensity, but as the PID particle number in a cell or a defined area in tissues. The fluorescence intensity of the PIDs changes depending on the specific configuration of the optical system. However, the particle number of PIDs is an absolute value and does not vary across systems. In this study, we obtained IHC-PIDs data by using primarily a normal fluorescence microscope manufactured by the Olympus Corporation. Because the number of PID particles per cell determined using the Zeiss and Keyence normal fluorescence microscopes was significantly correlated with the Olympus data, the IHC-PIDs method is a versatile optical technique that can estimate accurate protein expression levels independently of the optical system.

Figure 4 shows that the dynamic range of IHC-PIDs (10^−6^ to 10^−1^ mM) was 1,000-fold and more than 300-fold greater than that of DAB (10^−5^ to 10^−3^ mM) and a fluorescent probe (Texas Red, 10^−4^ to 3 × 10^−2^ mM; Qdots, 10^−4^ to 10^−2^ mM), respectively. PID imaging notably exhibited high sensitivity at low biotin concentrations. The high sensitivity over a greater dynamic range of IHC-PIDs is thought to be useful for accurate assessment of low-level biomolecular changes. During signal transduction, local modification or expression of proteins can play pivotal roles in important cell functions. Therefore, the detectability of target modifications or expression that occurs at low levels can have a strong effect on IHC-based studies and diagnoses.

PID staining enabled simultaneous imaging of cellular morphology and properties in a single tissue section stained with HE and PIDs (Fig. 6a–c). For the pathological section (2–4 μm thickness), we prepared two adjacent sections that were mirror images of each other. However, the thickness of the blade edge of the microtome used to prepare the section was approximately 1.5 μm, on the basis of the SEM image (data not shown). Therefore, even if two adjacent sections are mirror images, there is a gap of 1.5 μm between the two sections because this thickness of tissue is eliminated by the edge of the microtome. Therefore, it is challenging to observe an identical cross-sectional surface in two adjacent sections. Because we observed cellular morphology and properties in true identical sections using HE and PIDs staining, we believe that IHC-PIDs will be useful for performing cancer mechanism study and pathological diagnosis with high accuracy.

We successfully used IHC-PIDs of a biopsy sample to distinguish pCR and non-pCR of neoadjuvant chemotherapy with trastuzumab performed before chemotherapy and surgery. Knowledge of the diagnostic results before chemotherapy might allow doctors to modify the treatment regimen for patients who are expected to experience non-pCR by adding or substituting other HER2-targeted drugs with different mechanisms of action (e.g., pertuzumab^26^, T-DM1^27^, lapatinib^28^, or neratinib^28^). Therefore, we believe that IHC-PIDs might be used to select the anti-tumor effects of anticancer agents and thus benefit cancer patients. Additionally, because this new method enhances the therapeutic selectivity of drugs and promotes therapeutic efficiency of drug use, this method is expected to sharply reduce healthcare costs.

## Methods

### Preparation of perylene diimide assembly-conjugated nanoparticles

First, 16.5 mg of perylene diimide^29^ (Tokyo Chemical Industry, Tokyo, Japan) and 100 mg of Emulgen (Kao, Tokyo, Japan) were dissolved in 18.5 mL of distilled water. Then, the solution was stirred and heated at 70 °C. Next, 0.78 g of methylol melamine (Nippon Carbide Industries, Tokyo, Japan) and 980 μL of 10% dodecylbenzenesulfonic acid aqueous solution (Kanto Chemical, Tokyo, Japan) were added to the solution and heated at 65 °C for 30 min. After an additional 50 min after the 30 minutes at 65 °C, the solution was heated at 75 °C for 20 min. To wash the obtained perylene diimide-conjugated nanoparticles, the dispersion was centrifuged, and the supernatant was discarded. Distilled water was added to the sample and dispersed using a UH-50 ultrasonic homogenizer (SMT, Tokyo, Japan), and the sample was further washed using ethanol and distilled water. The obtained perylene diimide assembly-conjugated nanoparticles were then subjected to surface modification.

### Surface modification of perylene diimide assembly-conjugated nanoparticles

First, 20 mg of 1,2-bis(2-aminoethoxy)ethane (Tokyo Chemical Industry) was added to the dispersion of the perylene diimide assembly-conjugated nanoparticles and heated at 70 °C for 20 min to modify the amino groups. Next, 15 mg of NHS- PEG-MAL (Thermo Fisher Scientific, Tokyo, Japan) was added to the dispersion of the amino-modified perylene diimide assembly-conjugated nanoparticles and stirred at 20 °C for 1 h. To wash the obtained MAL-PEG-modified fluorescent nanoparticles, the dispersion was centrifuged at 10,000 × *g* for 20 min, and the supernatant was discarded. The sample was dispersed in distilled water using an ultrasonic homogenizer. 2-Iminothiolane HCl was added to 1 mL of a solution of 0.01 mg of streptavidin (Nacalai Tesque, Kyoto, Japan), stirred for 60 min, and then purified using a column to obtain thiolated streptavidin. The thiolated streptavidin was added to the dispersion of the MAL-PEG-modified perylene diimide assembly-conjugated nanoparticles to obtain streptavidin-modified fluorescent nanoparticles (PIDs). The dispersion was washed four times using PBS, and the PIDs were dispersed using 1% bovine serum albumin (BSA) in PBS. The number of streptavidin molecules on the PIDs was calculated from the streptavidin concentration measured using the BCA method (Pierce, Thermo Fisher Scientific, Tokyo, Japan) (Supplementary Fig. 1). The measurements indicated that the surface of a single PID was attached to approximately 2,460 streptavidin molecules via PEG chains.

### Measurement of the fluorescence properties of PIDs

The excitation and emission wavelengths of perylene diimide and PID particles in PBS were investigated using a spectrometer (F-7000, Hitachi, Tokyo, Japan). The 580 nm light-excited fluorescence intensity of perylene diimide, Qdot 625 (Thermo Fisher Scientific; the number indicates the emission wavelength) or PID particles was examined using an F-7000 spectrometer. The fluorescence intensities of a single molecule of perylene diimide, a single Qdot 625 particle, and a single PID particle were calculated and compared. To examine the photostability of the perylene diimide and PIDs, each was irradiated with 580 nm light, and the fluorescence intensities were measured over time using a BX53 fluorescence microscope (Olympus, Tokyo, Japan) with a UPLSAPO objective lens 40 × 2 (40×, 0.95 NA, Olympus) and a DP73 CCD camera (Olympus).

### Evaluation of the dynamic range of IHC-PIDs

To examine the dynamic range of IHC-PIDs, mimic slides coated with various concentrations of biotin were imaged. Paraffin sections of human breast cancer tissue (US Biomax, MD, USA) were deparaffinized in xylene and hydrated with a graded alcohol series and distilled water. The samples were incubated with 10^−6^, 10^−5^, 3 × 10^−5^, 10^−4^, 3 × 10^−4^, 10^−3^, 3 × 10^−3^, 10^−2^, 3 × 10^−2^, or 10^−1^ mM NHS-PEG-biotin (Thermo Fisher Scientific) at 25 °C for 1 h. This treatment caused the NHS-PEG-biotin to bind to the tissue sections. After a PBS wash, the samples were incubated with 1% BSA in PBS at 25 °C for 10 min for blocking. The samples were washed and treated with streptavidin-conjugated HRP (I-VIEW DAB Universal kit, Roche, Tokyo, Japan), 20 μg/mL streptavidin-conjugated Texas Red (Vector Laboratories, CA, USA), 40 nM streptavidin-conjugated Qdot 625 particles (Thermo Fisher Scientific) or 0.05 nM PIDs at 25 °C for 1 h. The concentrations of streptavidin-conjugated HRP, Texas Red, and Qdot 625 were as recommended in the manufacturer’s instructions. After being washed with PBS, all samples were dehydrated and mounted in mounting medium for observation. For this observation, the focal point was the interstitial area in the tissues, which were imaged with an optical microscope (BX53, Olympus) using a UPLSAPO 40 × 2 (Olympus) objective lens and CCD camera (DP73, Olympus). The sample stained with streptavidin-conjugated HRP was treated with DAB (I-VIEW DAB Universal Kit, Roche), observed with a bright field microscope, and analyzed using the Aperio image analysis system (Leica, Tokyo, Japan). The samples stained with streptavidin-conjugated Texas Red, streptavidin-conjugated Qdot 625 particles or PIDs were observed through a fluorescence filter (64HE, Zeiss, Tokyo, Japan). Four microscopic fields in each sample were observed, and the mean values were obtained.

### Preparation of paraffin sections using cultured human breast cancer cells

Cultured breast cancer cells (1.5 × 10^7^ cells) were fixed in 10% neutral buffered formalin mixed with alginic acid solution. The sample was embedded with alginate gel and treated with paraffin to prepare a paraffin block using a dedicated device (Retratome REM-700, Yamato Kohki, Saitama, Japan). The samples were cut into 3 μm thick sections and mounted on glue-coated glass slides.

### IHC of paraffin sections of cultured human breast cancer cells with PIDs

All paraffin sections were deparaffinized in xylene and hydrated in a series of graded alcohols and distilled water. Antigen retrieval was performed by boiling the samples in 10 mM citrate buffer (pH 6.0) for 40 min at 98 °C. Then, the samples were immunostained with the monoclonal antibody 4B5 (Roche) at 25 °C for 40 min. The samples were washed with PBS and then incubated with 6 μg/mL biotinylated secondary antibody. Before incubation with the secondary antibody, the anti-rabbit monoclonal antibody (LO-RG-1, Bio-Rad, CA, USA) was monomerized through a reduction reaction. The antibody monomerization exposed the thiol group of the hinge region in the antibody, which was biotinylated using Biotin Labeling Kit-SH (Dojindo, Kumamoto, Japan). After incubation with the biotinylated secondary antibody at 25 °C for 30 min, the sample was treated with 0.02 nM PID at 25 °C for 1 h. The sample was washed, stained with hematoxylin and mounted in mounting medium (multimount480, Matusnami, Osaka, Japan). The PID fluorescence signals were observed using fluorescence microscopy (BX53, Olympus) with a UPLSAPO 40 × 2 (Olympus) objective lens and CCD camera (DP73, Olympus) in 5 microscopic fields (with 1,000 cells investigated in each sample).

### Analysis of the HER2 expression level in human cultured cancer cells by FACS

Cultured breast cancer cells were fixed in 10% formalin, and the samples (2 × 10^6^ cells) were mixed with PBS containing 5% fetal bovine serum, 1 mM EDTA, and 0.1% NaN_3_. The samples were immunostained with 5 μg/mL primary antibody to HER2 (Anti-C-ErbB2/c-Neu(Ab-5) Mouse mAb(TA-1), Calbiochem, Merck Millipore, Tokyo, Japan) at 4 °C for 30 min. After a PBS wash, the samples were incubated with 1 μg/mL secondary antibody (Anti-Mouse IgG-Alexa Fluor488, Abcam, Tokyo, Japan) at 4 °C for 30 min. After a PBS wash, the samples were mixed with dedicated buffer for FACS and subjected to FACS measurements (MACSQuant Analyzer, Miltenyi Biotec, Bergisch Gladbach, Germany). We measured the fluorescence intensity per cell of 20,000 cells by FACS^19^ and calculated the mean value. Fluorescence-labeled beads (QIFIKIT) were also measured for calibration. QIFIKIT is used to determine the density of antibody-binding antigen per cell using FACS.

### Patients and breast tissue specimens

We identified 6,824 patients with primary invasive breast cancer who underwent surgery at the Department of Surgery, Tohoku University Hospital (Sendai, Japan), Iwate Prefectural Central Hospital (Morioka, Japan), Tohoku Kosai Hospital (Sendai, Japan), Miyagi Cancer Center Hospital (Natori, Japan), Nihonkai General Hospital (Sakata, Japan), Osaki Citizen Hospital (Osaki, Japan), or Japanese Red Cross Ishinomaki Hospital (Ishinomaki, Japan) between January 2007 and December 2013. We selected 74 HER2-positive breast cancer specimens from 89 patients who were treated with neoadjuvant chemotherapy with trastuzumab. The pathological response of the surgically resected tumor was evaluated after neoadjuvant chemotherapy. A pathological complete response (pCR) was defined as the absence of all invasive cancer cells and lymph node metastasis, regardless of the presence or absence of noninvasive cancer cells^25^. We used samples from 34 pCR and 40 non-pCR patients that were obtained from a biopsy before neoadjuvant chemotherapy.

### Ethics statement

The Ethical Committees of the Graduate School of Medicine at Tohoku University, Iwate Prefectural Central Hospital, Tohoku Kosai Hospital, Miyagi Cancer Center Hospital, Nihonkai General Hospital, Osaki Citizen Hospital, and Japanese Red Cross Ishinomaki Hospital approved this protocol. Before performing IHC and FISH in this study, we obtained informed consent from all the patients. All patients signed an Ethical Committee consent form and agreed to serve as tissue donors for the experiments. The methods were conducted in accordance with the approved guidelines.

### HER2 testing by IHC-DAB and FISH

HER2 testing by IHC-DAB was performed with a kit for IHC-DAB (HercepTest, Dako, CA, USA). The results of IHC-DAB were scored by two pathologists as follows: score 0 represents no staining or incomplete membrane staining in 10% or less of tumor cells; score 1 represents incomplete membrane staining in more than 10% of tumor cells; score 2 represents weak to moderate complete membrane staining in more than 10% of tumor cells or complete membrane staining in 10% or less of tumor cells; and score 3 represents intense circumferential membrane staining in more than 10% of tumor cells^22^. The DAB-stained sample was also analyzed with an Aperio image analysis system (Leica). FISH was conducted using a PathVysion HER2 DNA Probe Kit (Abbott, IL, USA). The slides were hybridized with probes to HER2/neu and the centromere marker CEP17. The ratio of HER2 genes to CEP17 genes was determined (ECLIPSE Ni, Nikon, Tokyo, Japan). A FISH score of 2.0 or greater indicated a HER2-positive sample.

### IHC of paraffin sections of human breast cancer patient tissues with PIDs

All breast tumor tissue specimens were fixed in 10% neutral buffered formalin and embedded in paraffin according to standard procedures. The samples were cut into 3 μm thick sections and placed on glue-coated glass slides. The subsequent processes were performed in the same manner as the IHC of the paraffin sections of cultured human breast cancer cells.

### Electron microscope

In this study, S-4800 (Hitachi) was used for scanning electron microscopy (SEM).

### Optical system

In this study, an Axio Imager Z1 (Zeiss) fluorescence microscope with a CCD camera (AxioCam, Zeiss), a BX53 microscope (Olympus) with a CCD camera (DP73 Olympus), and a BZ-X700 microscope (Keyence, Tokyo, Japan) with a CCD camera (BZ-X710, Keyence) were used for IHC. An ECLIPSE Ni microscope (Nikon) with a CCD camera (Ds-Qi1Mc, Nikon) was used for FISH.

### Statistical analysis

All data are presented as the mean ± s.e.m. *F*-tests were performed, and equal variance was defined as a *p*-value (*p*) ≥ 0.05. Comparisons between groups were performed using parametric Student’s *t*-tests (*p* ≥ 0.05 in the *F*-test) or Welch’s *t*-tests (*p* < 0.05 in the *F*-test). A value of *p* < 0.05 was considered significant for both *t*-tests.

## Electronic supplementary material

Supplementary Information
