# Supplementary material for: Quantitative diagnostic imaging of cancer tissues by using phosphor-integrated dots with ultra-high brightness
Source: Sci Rep. 2017 Aug 8;7:7509. doi: 10.1038/s41598-017-06534-z (PMC5548777; doi:10.1038/s41598-017-06534-z)
Supplement: Supplementary file 1 — Supplementary Information [file 41598_2017_6534_MOESM1_ESM.pdf]

Quantitative diagnostic imaging of cancer tissues by using phosphor-integrated dots with ultra-high brightness

Kohsuke Gonda[1,2,*], Mika Watanabe[3], Hiroshi Tada[4], Minoru Miyashita[4], Yayoi Takahashi-Aoyama[3], Takashi Kamei[5], Takanori Ishida[4], Shin Usami[6], Hisashi Hirakawa[7], Yoichiro Kakugawa[8], Yohei Hamanaka[4,9], Ryuichi Yoshida[10], Akihiko Furuta[11], Hisatake Okada[12], Hideki Goda[12], Hiroshi Negishi[12], Kensaku Takanashi[12], Masaru Takahashi[12], Yuichi Ozaki[13], Yuka Yoshihara[12], Yasushi Nakano[12], Noriaki Ohuchi[2,4].

[1]Department of Medical Physics, Graduate School of Medicine, Tohoku University, Seiryo-machi, Aoba-ku, Sendai 980-8575, Japan

[2]Department of Nano-Medical Science, Graduate School of Medicine, Tohoku University, Seiryo-machi, Aoba-ku, Sendai 980-8575, Japan

[3]Department of Pathology, Tohoku University Hospital, Seiryo-machi, Aoba-ku, Sendai 980-8574, Japan

[4]Department of Breast and Endocrine Surgical Oncology, Graduate School of Medicine, Tohoku University, Seiryo-machi, Aoba-ku, Sendai 980-8574, Japan

[5]Department of Gastroenterological Surgery, Graduate School of Medicine, Tohoku University, Seiryo-machi, Aoba-ku, Sendai 980-8574, Japan

[6]Department of Breast Surgery, Iwate Prefectural Central Hospital, Ueda, Morioka 020-0066, Japan

[7]Department of Breast Surgery, Tohoku Kosai Hospital, Kokubuncho, Aoba-ku, Sendai 980-0803, Japan

[8]Department of Breast Surgery, Miyagi Cancer Center Hospital, Medeshima, Natori 981-1293, Japan

[9]Department of Breast Surgery, Nihonkai General Hospital, Akiho-cho, Sakata 998-8501, Japan

[10]Department of Breast Surgery, Osaki Citizen Hospital, Furukawa, Osaki 989-6174, Japan

[11]Department of breast surgery, Japanese Red Cross Ishinomaki Hospital, Ishinomaki, Miyagi, 986-8522, Japan

[12]Bio Health Care, Business Development Division, Business Development Headquarters, Konica Minolta, Inc., No. 1 Sakura-machi, Hino-shi Tokyo 191-8511, Japan

[13]Data Science Center, Systems Technology Division, Business Development Headquarters, Konica Minolta, Inc., No. 1 Sakura-machi, Hino-shi Tokyo 191-8511, Japan

[*]Correspondence should be addressed to K.G. (gonda@med.tohoku.ac.jp)

Supplementary figure, figure legend, table, table legend, and reference

Supplementary figure and figure legend

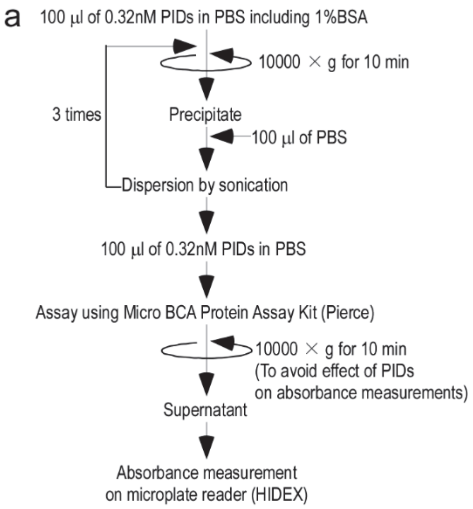

b

Lot no. of PIDs	PIDs conc. (nM)	Streptavidin conc. (μg/ml)	Number of streptavidin molecules on the suface of single PID
1	0.32	40.7	2420
2	0.23	29.8	2430
3	0.24	37.4	2540

Supplementary Figure 1.

Measurement of the number of streptavidin molecules on the surfaces of PIDs. The PIDs had streptavidin attached via the terminal site of the PEG chains at the surface (Fig. 1a). The number of streptavidin molecules was tested using a kit (**a**). First, to remove BSA from the sample, 100 μL of 0.32 nM PID in PBS including 1% BSA was centrifuged at 10,000 ×*g* for 10 min, and the supernatant was discarded. The precipitate was dispersed in 100 μL of PBS using sonication (**a**). This process was repeated three times. Then, the streptavidin concentration was measured using a Micro BCA Protein Assay Kit (Pierce) according to the manufacturer's instructions (**a**). To avoid the effect of PIDs on absorbance measurements, the solution obtained after the reaction using the above kit was centrifuged at 10,000 ×*g* for 10 min, and the supernatant was used for the absorbance measurement on a microplate reader (**a**). The resulting data were similar for three repetitions of the experiment and demonstrated that a single PID was coated with approximately 2,460 streptavidin molecules via the terminal sites of the PEG chains on its surface (**b**).

Supplementary table and table legend

Supplementary Table 1. Results of four diagnostic scores for HER2 expression, DAB score, DAB intensity, FISH score and PID score for 74 patients treated with neoadjuvant chemotherapy with trastuzumab.

Patient No.	pCR /non-pCR	Age	cT	cN	cStage	DAB score	DAB intensity	FISH score	PID score
1	pCR	57	2	0	IIA	3	168.5	6.3	96.6
2	pCR	63	4	3	IV	3	98.4	4.3	38.9
3	pCR	63	2	0	IIA	3	119.3	n/a	103.6
4	pCR	30	2	1	IIB	3	92.2	3.9	76.7
5	pCR	60	4	3	IIIC	3	90.5	n/a	47.1
6	pCR	63	2	1	IIB	3	106.0	n/a	77.2
7	pCR	65	2	1	IIB	3	84.2	3.4	29.6
8	pCR	44	1	1	IIA	3	115.3	5.0	55.0
9	pCR	57	1	1	IIA	3	72.7	5.0	53.1
10	pCR	60	2	1	IIB	3	136.3	3.2	62.0
11	pCR	63	2	1	IIB	3	124.0	4.0	59.1
12	pCR	61	2	1	IIB	3	98.4	6.1	61.5
13	pCR	69	2	1	IIB	3	119.5	3.6	63.4
14	pCR	47	3	1	IIIA	2	105.0	3.0	20.0
15	pCR	40	2	0	IIA	2	88.6	3.0	25.0
16	pCR	61	2	1	IIB	3	161.0	n/a	153.4
17	pCR	55	2	1	IIB	3	162.0	n/a	67.5
18	pCR	56	2	1	IIB	3	159.2	2.9	54.1
19	pCR	32	1	0	I	3	175.3	4.0	64.2
20	pCR	48	3	1	IIIA	3	153.9	4.0	88.6
21	pCR	34	2	3	IIIC	3	147.4	5.1	84.4
22	pCR	57	2	1	IIB	3	141.9	7.8	67.8
23	pCR	52	2	1	IIB	3	117.0	3.7	30.1
24	pCR	55	2	1	IIB	3	142.7	5.0	84.1
25	pCR	56	3	3	IIIC	3	119.5	4.6	76.5
26	pCR	51	1	2	IIIA	3	149.3	4.2	67.2

27	pCR	43	2	1	IIB	3	120.3	3.2	14.2
28	pCR	70	4	3	IIIC	3	167.8	5.9	63.9
29	pCR	61	4	1	IIIB	3	139.7	5.1	48.7
30	pCR	52	4	1	IIIB	3	154.4	5.1	63.4
31	pCR	68	4	2	IIIB	3	148.1	4.8	76.4
32	pCR	71	2	2	IIIA	3	154.5	2.9	80.8
33	pCR	41	4	3	IIIC	3	100.7	5.2	26.3
34	pCR	67	2	1	IIB	3	111.5	3.5	74.6
35	non-pCR	62	4	3	IIIC	3	146.6	3.2	47.9
36	non-pCR	44	4	1	IIIB	3	151.4	5.1	79.6
37	non-pCR	50	2	1	IV	3	125.3	3.1	54.0
38	non-pCR	57	2	1	IIB	2	61.2	2.7	10.4
39	non-pCR	49	2	1	IIB	3	148.2	8.0	78.0
40	non-pCR	61	3	3	IIIC	3	104.8	4.9	66.0
41	non-pCR	42	4	3	IIIC	3	68.9	4.3	27.6
42	non-pCR	54	1	3	IIIC	3	94.9	4.2	37.5
43	non-pCR	58	1	3	IV	3	59.6	4.7	44.0
44	non-pCR	49	2	0	IIA	3	118.5	7.2	73.8
45	non-pCR	54	4	3	IV	3	128.3	6.0	43.1
46	non-pCR	60	4	3	IV	2	55.4	2.0	3.5
47	non-pCR	36	2	1	IIB	3	150.9	8.4	36.7
48	non-pCR	65	3	0	IIB	3	108.2	7.4	26.4
49	non-pCR	45	2	1	IIB	3	132.0	5.7	78.5
50	non-pCR	39	2	0	IIA	3	134.3	6.6	38.9
51	non-pCR	79	2	1	IIB	3	131.2	3.5	66.2
52	non-pCR	72	4	3	IIIC	1	118.4	3.8	80.6
53	non-pCR	57	2	0	IIA	3	111.1	3.5	33.0
54	non-pCR	54	4	1	IIIB	3	120.9	n/a	55.9
55	non-pCR	56	2	0	IIA	3	168.5	2.9	80.8
56	non-pCR	61	2	3	IIIC	3	145.8	4.8	69.5
57	non-pCR	36	3	2	IIIA	3	174.6	5.8	84.9
58	non-pCR	39	2	1	IIB	3	139.0	5.8	63.9
59	non-pCR	54	2	3	IIIC	2	96.7	4.0	40.7

60	non-pCR	61	4	1	IIIB	3	109.1	4.4	31.8
61	non-pCR	43	2	1	IIB	3	171.6	2.9	83.7
62	non-pCR	40	2	1	IIB	2	93.9	4.4	39.7
63	non-pCR	34	3	1	IIIA	3	163.2	3.7	93.1
64	non-pCR	77	2	1	IIB	3	162.3	4.3	78.6
65	non-pCR	37	2	0	IIA	3	137.7	6.1	53.7
66	non-pCR	53	4	1	IIIB	2	106.2	3.8	19.7
67	non-pCR	49	2	2	IIIA	3	89.1	6.2	24.3
68	non-pCR	59	2	3	IIIC	2	98.2	4.8	42.8
69	non-pCR	52	4	2	IIIB	2	74.4	6.6	15.9
70	non-pCR	32	2	1	IIB	3	110.0	2.7	53.1
71	non-pCR	58	1	0	I	2	55.7	2.0	4.3
72	non-pCR	58	4	1	IIIB	3	117.7	3.2	31.1
73	non-pCR	56	3	1	IIIA	3	148.5	4.4	56.9
74	non-pCR	51	4	3	IIIC	3	116.4	7.8	48.8

pCR: pathological complete response, cT: clinical T category, which describes the primary tumor size, cN: clinical N category, which describes the lymph node involvement, cStage: clinical stage grouping, which is based on the TNM Classification of Malignant Tumors Seventh Edition by the International Union Against Cancer (UICC)[30], DAB score: HercepTest score (scores of 0, 1, 2, and 3), DAB intensity: staining intensity of IHC-DAB analyzed using Aperio image analysis, FISH score: ratio of HER2 genes to CEP17 genes, PID score: number of PID particles per 100μm^2, n/a: not available.

Supplementary Reference

30. L.H. Sobin, M.K. Gospodarowwicz, C. Wittekind, TNM Classification of Malignant Tumours 7th editon. John Wiley & Sons, New Jersey, USA, (2009) pp.181–193.
